# Development and validation of a semi-automated measurement tool for calculating consistent and reliable surface metrics describing cosmesis in Adolescent Idiopathic Scoliosis

**DOI:** 10.1038/s41598-023-32614-4

**Published:** 2023-04-05

**Authors:** Sinduja Suresh, Pasan Perera, Maree T. Izatt, Robert D. Labrom, Geoffrey N. Askin, J. Paige Little

**Affiliations:** 1 Biomechanics and Spine Research Group (BSRG), Centre for Biomedical Technologies (CBT) at the Centre for Children’s Health Research (CCHR), School of Mechanical Medical and Process Engineering, Queensland University of Technology, Brisbane, Australia; 2 Orthopaedics Department, Queensland Children’s Hospital (QCH), Brisbane, Australia

**Keywords:** Biomedical engineering, Paediatric research, Skeleton, Quality of life, Three-dimensional imaging

## Abstract

Adolescent Idiopathic Scoliosis (AIS) is a 3D spine deformity that also causes ribcage and torso distortion. While clinical metrics are important for monitoring disorder progression, patients are often most concerned about their cosmesis. The aim of this study was to automate the quantification of AIS cosmesis metrics, which can be measured reliably from patient-specific 3D surface scans (3DSS). An existing database of 3DSS for pre-operative AIS patients treated at the Queensland Children’s Hospital was used to create 30 calibrated 3D virtual models. A modular generative design algorithm was developed on the Rhino-Grasshopper software to measure five key AIS cosmesis metrics from these models—shoulder, scapula and hip asymmetry, torso rotation and head-pelvis shift. Repeat cosmetic measurements were calculated from user-selected input on the Grasshopper graphical interface. InterClass-correlation (ICC) was used to determine intra- and inter-user reliability. Torso rotation and head-pelvis shift measurements showed excellent reliability (> 0.9), shoulder asymmetry measurements showed good to excellent reliability (> 0.7) and scapula and hip asymmetry measurements showed good to moderate reliability (> 0.5). The ICC results indicated that experience with AIS was not required to reliably measure shoulder asymmetry, torso rotation and head-pelvis shift, but was necessary for the other metrics. This new semi-automated workflow reliably characterises external torso deformity, reduces the dependence on manual anatomical landmarking, and does not require bulky/expensive equipment.

## Introduction

Adolescent Idiopathic Scoliosis (AIS) is the most common type of scoliosis^1^ and has been reported to affect 0.93–12% of adolescents (10–18 years old) worldwide^2–4^, depending on the region. It is a complex three dimensional spine deformity characterised by a lateral curvature of the spine, loss of normal sagittal curves and is accompanied by a rotation of the spine and ribcage^5–7^. This results in an externally observable torso distortion which affects normal posture^8^. While clinical metrics describing AIS like the Cobb angle^9^ and Angle of Torso Rotation (ATR) or rib hump^10^ are important for surveillance, management and treatment of the condition, what is often most important to the patients (and their families) is their cosmesis, or “how they look”^11^. Cosmetic improvement has been shown to directly influence patient quality of life^12,13^, and correcting the aesthetics of the torso is therefore a prime consideration when treating AIS^14^. Consequently, significant effort has been invested into developing metrics that characterise the posterior surface of the torso.

The quantification of surface topography (ST) in AIS has evolved from the subjective scoring of diagrams and photographs^15–22^ to objective methods like the Moiré technique^23,24^, rasterstereography^25–31^, photogrammetry^32–34^, depth sensors^35,36^ and infrared thermography^37^ that generate cosmesis metrics and indices. Limitations of current methods include complicated patient positioning and the use of ambiguous anatomical landmarks which are the major sources of measurement errors^38^. Further, advanced instruments for surface topography measurements are either too bulky to install, too expensive to purchase and maintain, require trained technicians to operate, involve indirect/complex/specific interpretations, or all the above^38–40^. It is important to note that while an increase in measurement accuracy is always desired, not every clinic or hospital can easily adopt these technologies into regular practice.

A review of existing literature highlights the need for a 360° 3D cosmesis assessment method that is simple, accurate, reliable, automation friendly and easily translatable into the clinical pathway. As a step in this direction, 3D surface scanning (3DSS) has garnered significant interest within the scoliosis community in recent years. Accuracy, reliability and validity have been investigated for full body scanners^41–43^, multi-component scanning systems^44–51^ and handheld scanners^52–54^ and the resultant reconstructed 3D Computer Aided Design (CAD) models have begun to be used to assess AIS cosmesis. Of these, portable handheld scanners are of particular interest as they have a high geometric accuracy^53^ and can potentially be widely and easily adopted into regular clinical practice and telehealth initiatives.

So far, 3DSS data from handheld scanners have predominantly been used to perform markerless symmetry analysis of the torso. This is done by estimating a sagittal plane of symmetry on the torso, separating the halves, creating mirrored models, and then performing a 3D deviation analysis on the superimposed original and mirrored halves^46–48,52^. While markerless symmetry analysis is an adequate method for identifying and tracking overall AIS progression, there is still scope for the quantification of individual metrics of spine deformity that are currently mostly visually assessed by treating clinicians.

This study aims to make a major contribution to this new and growing area of AIS research by using 3DSS to make reliable objective measurements of five key AIS cosmetic features. To this end, we have developed a user-friendly semi-automated measurement tool to standardise and streamline this process. The reliability study presented here tests the agility of the tool and ascertains how much experience is required to use it effectively to assess trunk deformity parameters in AIS.

## Methods

### Preliminary data processing

An existing database of 3DSS data for AIS patients treated at the Queensland Children’s Hospital Spine Clinic (QCHSC) was used to create 30 calibrated pre-operative patient-specific 3D virtual models. The dataset included patients with Lenke curve types (type 1 = 17, type 2 = 1, type 3 = 5, type 4 = 1, type 5 = 5, type 6 = 1) and both sexes (male = 2, female = 28). The mean age was 14.3 years (range 11.3–17 years), mean Body Mass Index (BMI) was 18.08 kg/m^2^ (range 14.4–25.6 kg/m^2^), mean Cobb angle of the major curve was 63° (range 38–105°) and mean Angle of Torso Rotation (ATR) of the major curve was 20° (range 12–38°).

**Fig 1:**
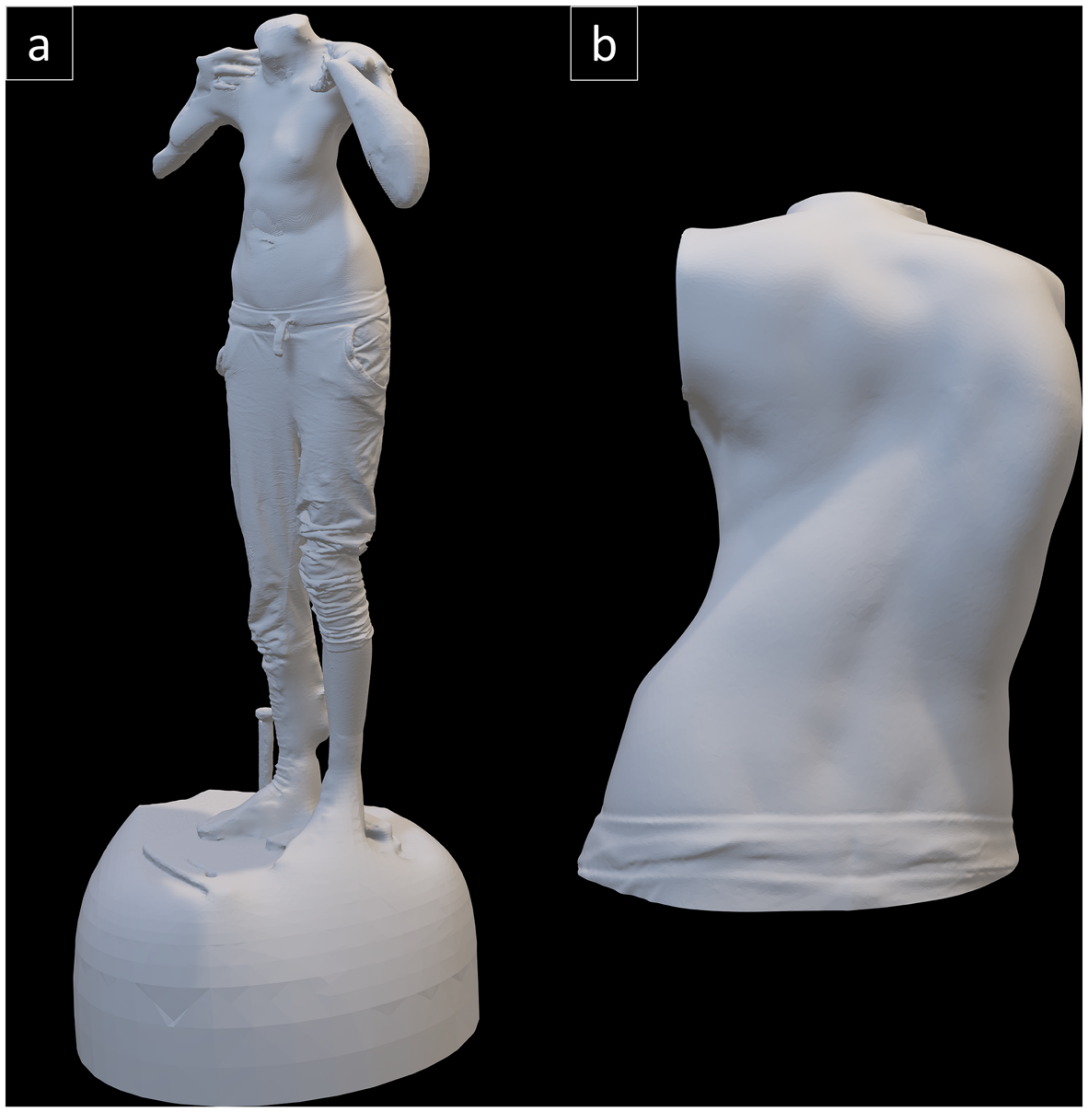
Preliminary data processing. (**a**) Stitched scan obtained from Artec, (**b**) Fully processed model obtained from Geomagic.

Patients were scanned at 16 frames per second using the Artec Eva white light scanner (Artec Group Inc., Luxembourg) in the standing position with fingertips placed on their shoulders. Each scan takes roughly a minute. The raw scans were stitched in the associated software Artec Professional 10, and the resultant 3D models were then exported in binary stereolithography (.stl) format to be processed using a standardised workflow in Geomagic Wrap (2020, 3D Systems, North Carolina) developed in-house. Here the models were aligned to the global coordinate system using the standardised coordinate system of the base wooden board on which the patients stood to be scanned. The models were subsequently cleaned of breathing artefacts and any unwanted items captured in the background, and cropped to only include the torso (head and limbs were removed). The torso model was then globally smoothed once using the “Quick Smooth” function and the “Mesh Doctor” tool was used to repair imperfections in the polygon mesh. Excessive smoothing and noise reduction can reduce detail in the mesh, so global smoothing was only performed once per model. The mesh was then conservatively decimated to reduce the file size. An example of a fully processed model is shown in Fig. 1.

### Metrics definition

Current practice at the QCHSC for cosmesis evaluation includes a detailed visual assessment of the patient which is recorded as a deformity description and a freehand scoliosis drawing in a spinal deformity assessment proforma. The deformity description identifies shoulder asymmetry, scapula prominence, loin fullness and head-pelvis shift in either the left or right side of the torso, but no objective ST measurements are included here. The ATR is measured later in the proforma with a Scoliometer tool while the patient is in forward flexion position (Adams Forward Bend) which acts as a proxy for the trunk rotation portion of scoliosis. While identifying key cosmetic asymmetries is sufficient for screening purposes, measuring their magnitude would allow clinicians to track external deformity progression alongside their surveillance of internal spine curve progression (measured from radiographs).

The Society of Scoliosis Orthopaedic and Rehabilitation Treatment (SOSORT) 6th consensus^55^ recommends measuring the back surface of the patient when in the standing position where the deformity is subject to the effects of gravity. Surface parameters recommended for systematic use include shoulder, scapulae and waist measurements, trunk rotation (main and compensatory curve) and Posterior Superior Iliac Spine (PSIS) height and depth, among others.

**Fig 2:**
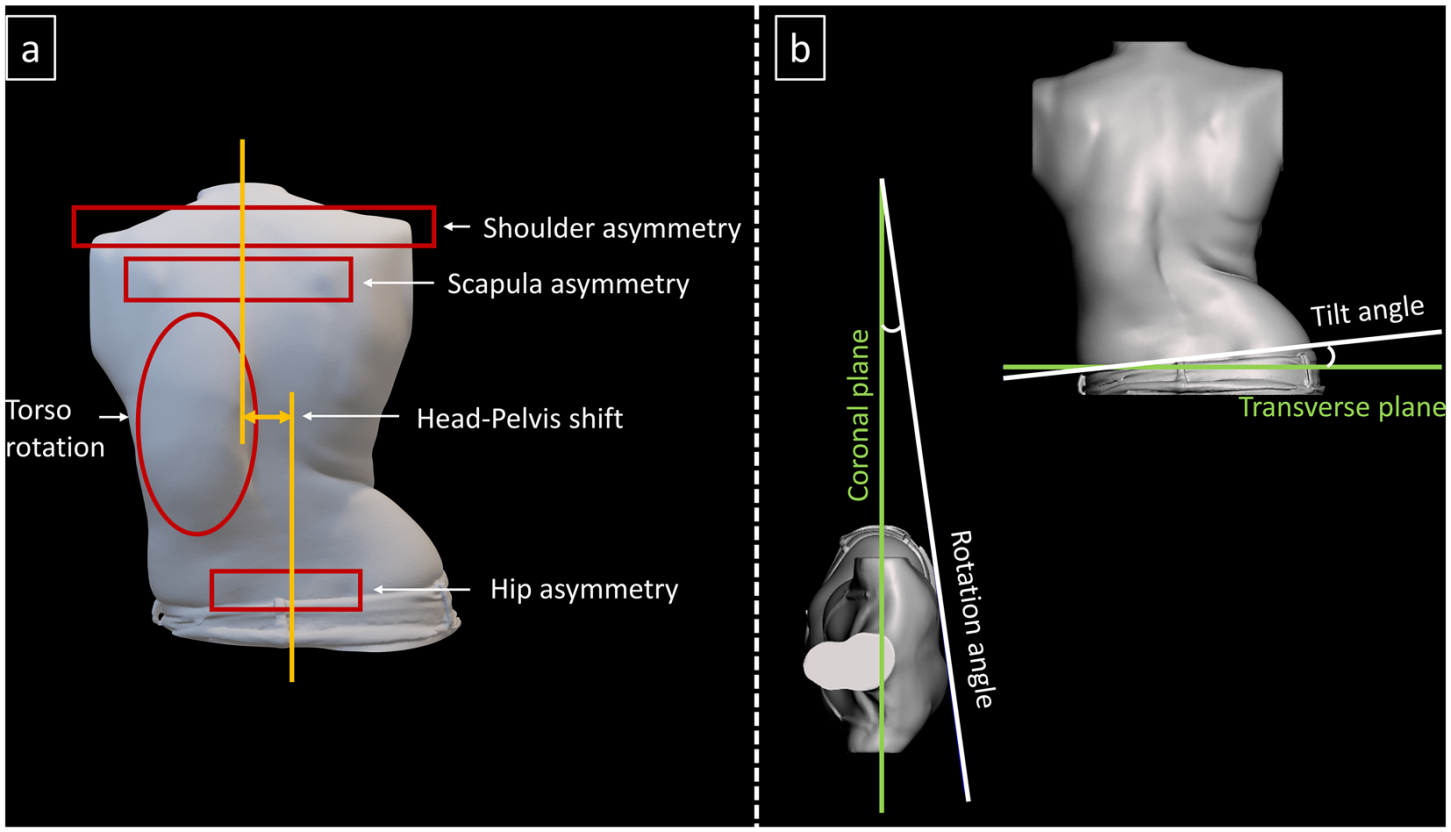
Metrics definition. (**a**) 5 key AIS metrics measurement regions, (**b**) Rotation and tilt definitions.

Therefore, five key AIS cosmesis metrics (Fig. 2a) were defined in accordance with current QCHSC practices and SOSORT guidelines—shoulder asymmetry which includes shoulder rotation (ShR) and shoulder tilt (ShT), scapulae asymmetry which includes scapulae rotation (ShR) and scapulae tilt (ShT), hip asymmetry which includes hip rotation (HR) and hip tilt (HT), torso rotation (TR) and head-pelvis shift (HPS). “Rotation” is defined as the angle deviation from the coronal plane (Fig. 2b) and “tilt” is defined as the angle deviation from the transverse plane (Fig. 2b).

### Algorithm development

A generative design parametric algorithm to measure cosmetic features was developed on the 3D modelling and graphical programming software Rhino 7 with Grasshopper add-on (Robert McNeel and associates, USA). The 3D mesh model can be manipulated on Rhino while user inputs to the algorithm are accepted through the Grasshopper interface. The user identifies regions of interest (ROI) that encapsulate the particular part of the anatomy corresponding to the region of cosmetic deformity. They then use a pre-defined volumetric primitive to encapsulate this area, allowing the developed algorithm to automatically identify key geometric features which correspond to what we see as a visual cosmetic deformity or feature. These features are based on both linear and angular measures as well as 3D spatial coordinates. Number sliders are displayed in the Grasshopper workflow which provide a dynamic user interface control of the ROIs. Individual algorithm components were clustered and segregated into 6 modules, one for the mesh input and five to measure each cosmesis metric for AIS.

### Measurement protocol

Two users, an “expert” user and a “novice” user, measured the AIS cosmesis metrics on the 3DSS data using the semi-automated measurement tool. The expert user was experienced in the Rhino-Grasshopper software and was knowledgeable about AIS (not a clinician), while the novice user had never used the software before and had no prior knowledge of AIS. Three measurement attempts by the expert and novice are denoted as E1, E2, E3 and N1, N2, N3 respectively. Both users were instructed to complete the measurements for the entire set of thirty patients before repeating them two more times to mitigate memory bias. Measurements took approximately 7 min to perform for all 5 metrics for one AIS patient.

### Statistical analysis

Reliability analysis was performed on R statistical computing software (RStudio 1.3.1093, R Foundation for Statistical Computing, Vienna, Austria)^56^ using a two-way, random effects, absolute agreements, single rater Inter-Class Correlation (ICC) according to the McGraw and Wong classification. Both intra-user (using 3 measurement sets from each user) and inter-user (using the third measurement set from each user) reliability were calculated.

ICC values of 0.5 and above indicate that the method is reliable. In detail, poor reliability is indicated by values less than 0.5, moderate reliability is indicated by values between 0.5 and 0.75, good reliability is indicated by values between 0.75 and 0.9, and excellent reliability is indicated by values greater than 0.90.

Sample size was calculated with a minimum accepted ICC reliability of 0.5 and an expected reliability of 0.8. Significance level was set at 0.05 (two-tailed) at a power of 80%^57,58^.

### Ethics statement

This research involves the analysis of existing anonymised and deidentified 3DSS data which were collected from patients as part of their standard clinical management at the QCHSC. Hospital and University Human Research Ethics approvals were obtained from QCH (HREC: LNR/21/QCHQ/75,249) and QUT (Approval number: 4856—HE44) titled “Spine Deformity Management Clinical Data Collection Project”. Informed consent was obtained from the patients and their legal guardians for their data to be included in this work. Approval to publish de-identified group data analyses by the QCH Human Research Ethics Committee (HREC) for the “Development of non-invasive monitoring tools for Adolescent Idiopathic Scoliosis, using 3D scanning/photography at the Queensland Children’s Hospital” was also provided.

The methods described in this paper are in accordance with the relevant guidelines and regulations put forth by the QCH HREC and the Declaration of Helsinki.

## Results

### Semi-automated measurement tool for AIS cosmesis metrics

**Fig 3:**
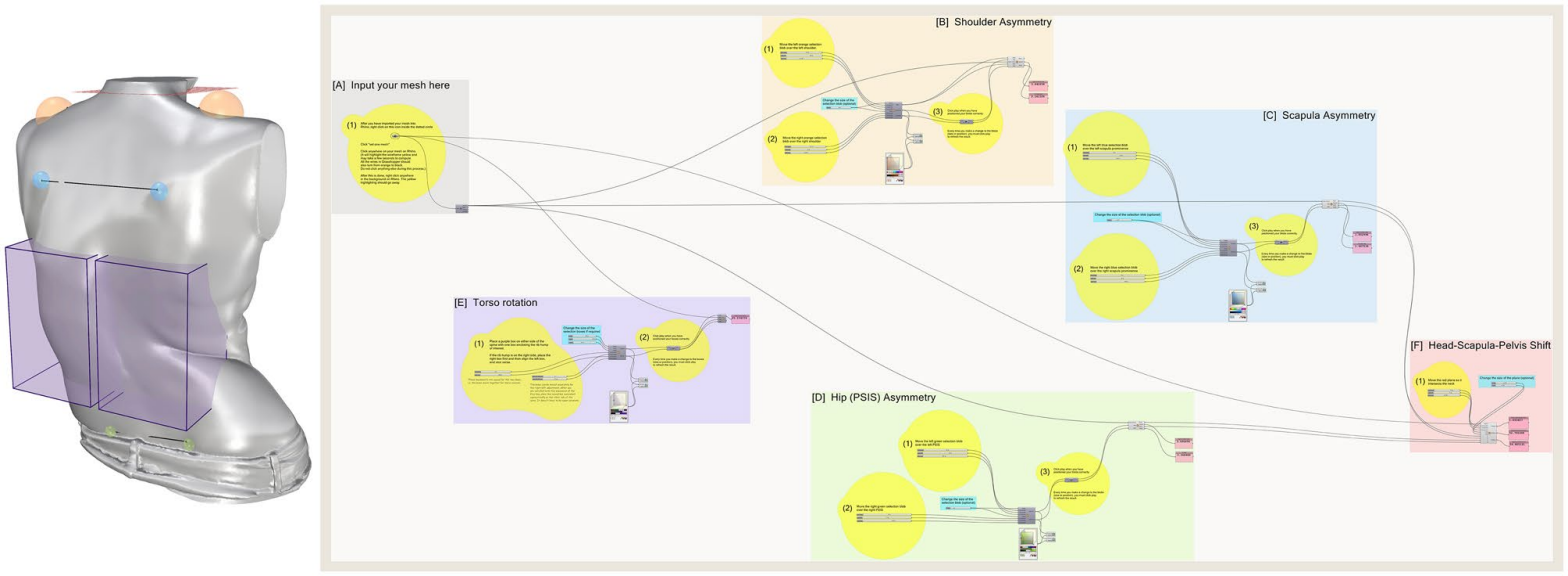
Rhino viewport (left) with Grasshopper user interface (right). The Rhino viewport shows a processed 3D model of an AIS patient (Lenke type 5). ROIs are shown placed in the correct positions for measurement. The Grasshopper modules are colour matched to the ROIs for each anatomical feature visible in the Rhino viewport—input (grey), shoulder (orange), scapula (blue), hip (green), torso rotation (purple), head-pelvis shift (red).

A semi-automated tool to measure five key AIS cosmesis metrics was successfully developed. The Rhino viewport and Grasshopper user interface are shown in Fig. 3.

The Grasshopper interface has 6 modules that are colour matched to the ROIs for each anatomical feature visible in the Rhino viewport (orange spheres for shoulder measurements, blue spheres for scapula measurements, green spheres for hip measurements, purple boxes for torso rotation measurements and a red plane for head-pelvis shift measurements).

Each module has 3 basic colour coded components. The yellow components require input from the user and contain sliders to move the ROIs in the 3D space, the blue components contain sliders to change the size of the ROIs if necessary, and the pink components display the calculated angle values. All other algorithm parts are clustered into small grey components which the user need not interact with. The functionality of each module is described below in detail.

#### Module [A]: Input

Any input mesh can be assigned to this module by dragging the previously created 3D mesh file (.stl format) into the Rhino workspace. This input mesh model will then be used for further calculations.

#### Modules [B], [C], and [D]: Shoulder, Scapula and Hip asymmetry (ShA, ScA, HA)

**Fig 4:**
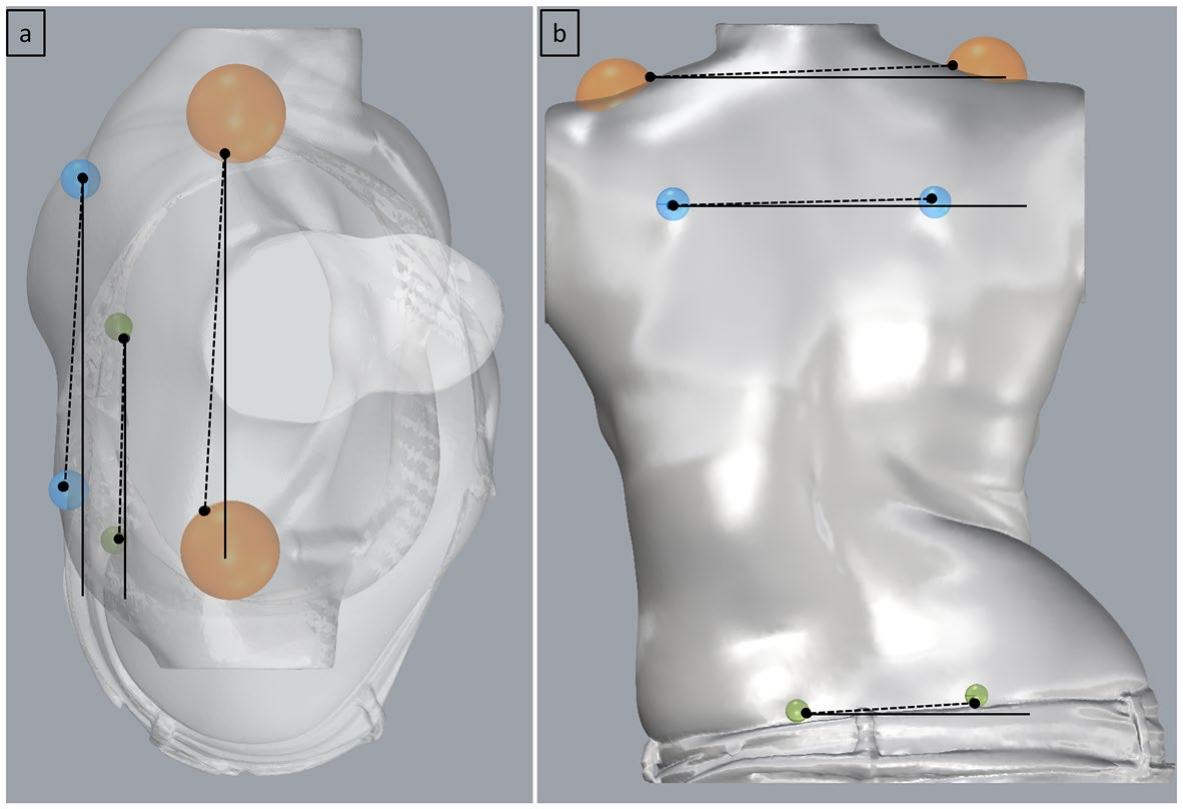
(**a**) rotation angle measurements of shoulder, scapula and hip (top view) and (**b**) tilt angle measurements of shoulder, scapula and hip (back view).

These three modules work in the same way. The user is able to drag a spherical region of interest (ROI) to mark the anatomical surface feature being measured. The ROI size can be changed within a small range to accommodate for patient variability. The algorithm then automatically calculates the angle of rotation and the angle of tilt of the anatomical feature being measured. For example, hip asymmetry is measured by calculating the angle between the left and right Posterior Superior Iliac Spine (PSIS) dimples. To achieve this, two ROIs are placed over the region of the PSIS dimples and their diameter modified by the user to ensure the ROI just encapsulates the surface depression on the skin. The most inward point in each PSIS dimple ROI is automatically calculated by the algorithm which then creates a line between these two points. The angle deviation of this line from the coronal (rotation) and transverse (tilt) planes are measured, shown in Fig. 4. Both the coronal and transverse planes (from which deviation is measured) are oriented to the world coordinate system.

#### Module [E]: Torso rotation (TR)

**Fig 5:**
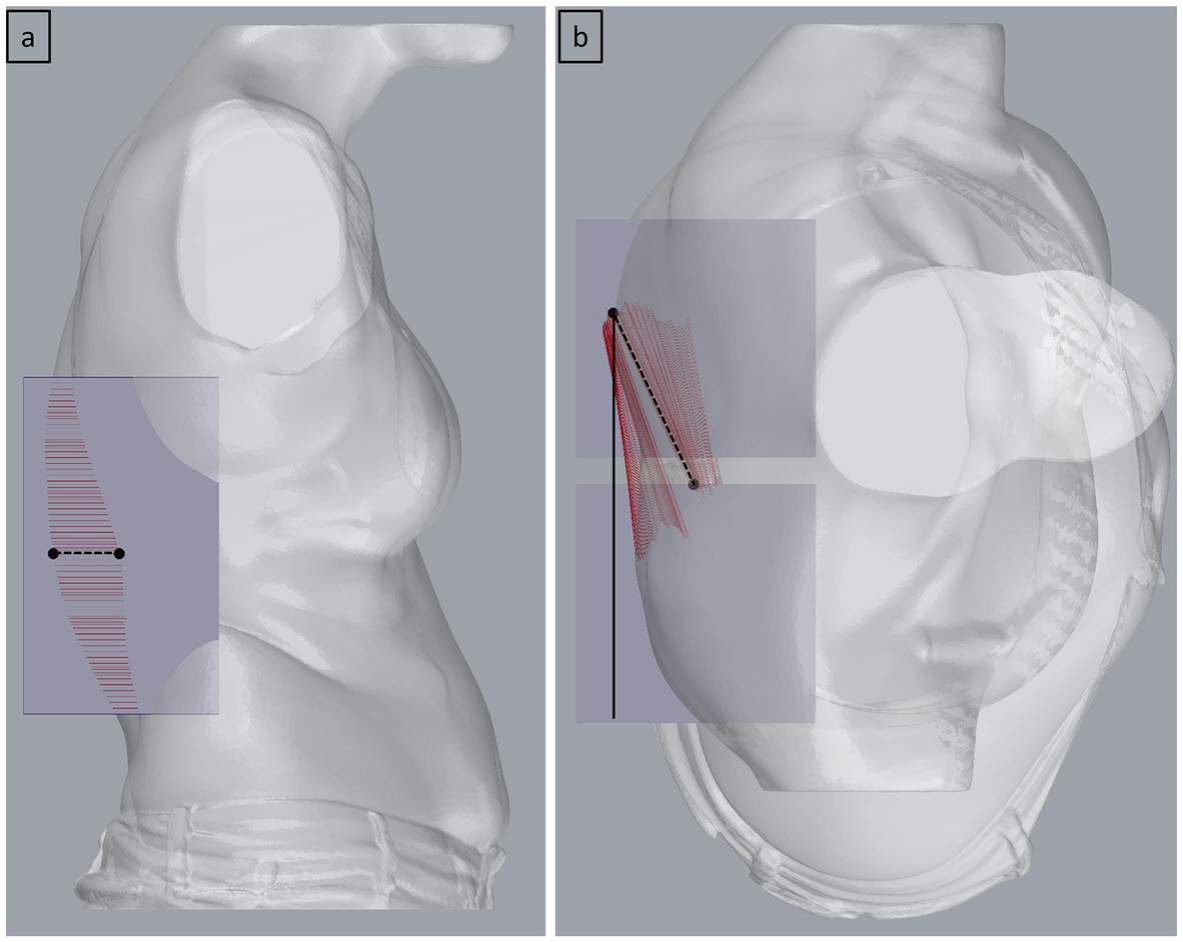
Torso rotation angle measurement (**a**) right view (**b**) top view.

The user places two cuboidal ROIs on each side of the spine to encapsulate the ribcage prominence. The algorithm then calculates the most outward point within each ROI vertically along the unit Z vector every 1 mm (this is the step resolution and can be changed if required). A line is drawn between the pair of extreme points at every step and deviation from the coronal plane is calculated for each line, replicating a scenario where an angle measurement is taken every 1 mm from the top to the bottom of the rib prominence when the patient is standing. The algorithm then identifies the line that has the maximum angle of deviation from the coronal plane. This is the angle of torso rotation (in the standing posture) measured from the ribcage prominence, imaged in Fig. 5.

#### Module [F]: Head-Pelvis Shift (HPS)

This module measures the lateral deviation of the head from the pelvis. The user is only required to move a small XY plane to intersect the neck. The algorithm then finds the centroid of this cross-section and calculates the lateral distance from there to the midpoint of the PSIS asymmetry line.

### Measurement reliability

**Fig 6:**
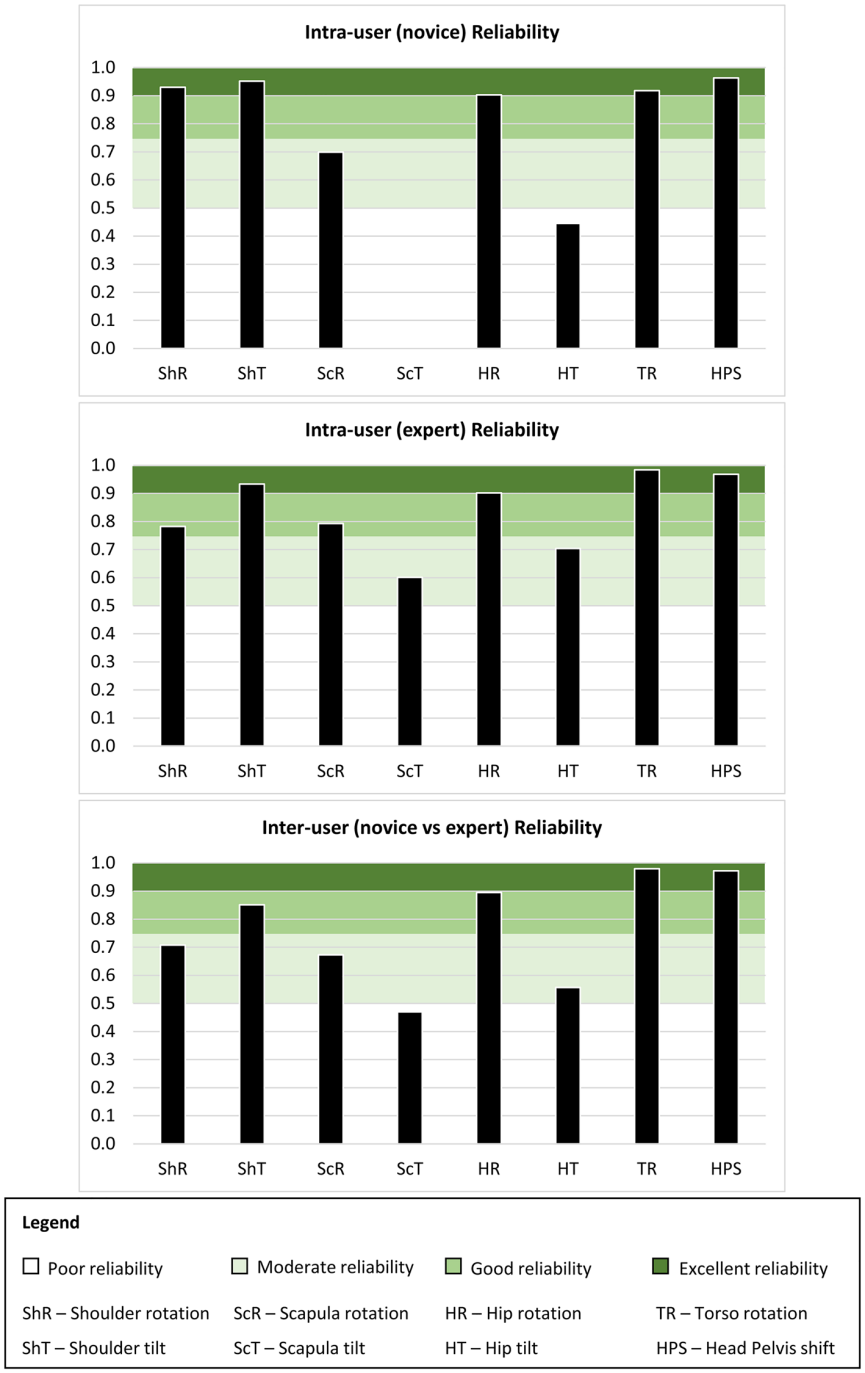
ICC reliability scores for intra-user and inter-user conditions.

Cosmetic measurements were calculated using the semi-automated measurement tool outlined above for the Rhino-Grasshopper graphical interface. The inter-class correlation (ICC) coefficients obtained from analysing the raw measurements are illustrated in Fig. 6.

Intra-user reliability for novice user measurements of shoulder asymmetry (ShA) was excellent (rotation = 0.93, tilt = 0.952), scapula asymmetry (ScA) was poor to moderate (rotation = 0.699, tilt = 0.003), hip asymmetry (HA) was poor to excellent (rotation = 0.903, tilt = 0.445), torso rotation (TR) was excellent (0.918) and head-pelvis shift (HPS) was excellent (0.963).

Intra-user reliability for expert user measurements of ShA was good to excellent (rotation = 0.782, tilt = 0.933), ScA was moderate to good (rotation = 0.793, tilt = 0.601), HA was moderate to excellent (rotation = 0.902, tilt = 0.704), TR was excellent (0.984) and HPS was excellent (0.968).

Inter-user reliability was excellent for TR (0.98) and HPS (0.972), moderate to good for ShA (rotation = 0.708, tilt = 0.851), poor to moderate for ScA (rotation = 0.673, tilt = 0.47) and moderate to good for HA (rotation = 0.895, tilt = 0.557).

The mean absolute difference between TR measurements of both users was 1.2 ± 0.7°.

## Discussion

Historically, ST analysis methods were first developed to reduce radiation exposure and associated cancer risks^59^ in AIS patients. With the advancement of low dose X-Ray and biplanar imaging systems in addition to the generally improved efficiency of radiographical procedures, AIS patients in the modern era now receive a long-term radiation dose below the carcinogenic threshold^60^. Regardless, it is still prudent to reduce radiation exposure wherever possible, especially in the growing child or adolescent. Additionally, it is well known that external torso distortion is at best only weakly correlated with the lateral curvature of the spine and that the Cobb angle is a single-plane measure that only describes one aspect of the 3D deformity of AIS^38^. The current value of ST analysis is, therefore, not only in reducing radiation exposure, but in providing quantitative information that is complementary to radiographic data^61^. The goal of this study was not to replace radiographical imaging of AIS, but to augment it with measurable metrics that are currently being qualitatively assessed by clinicians caring for adolescents with scoliosis.

Externally visible deformity is readily observed and is something that patients, parents and clinicians see changing as the spine deformity progresses. It is also an important factor to consider when assessing clinical outcomes of management techniques as there is an obvious improvement in cosmesis after corrective surgical treatment. While the Scoliosis Research Society (SRS) and SOSORT identify a reduction in Cobb angle as a primary treatment outcome and therefore a measure of success of a treatment method, SOSORT ranks the Cobb angle 8^th^ in treatment priorities after aesthetics, quality of life, disability, back pain, psychological well-being, progression in adulthood and long-term breathing function^4,14,62,63^. The objective tracking and measurement of torso aesthetics is therefore a very important part of patient-focussed care and holistic health management.

As seen in the literature, there is no focus on developing indices that can be clearly interpreted by clinicians and patients alike to describe AIS cosmetic deformity. There is still a large gap between ST metrics development and actual real-world applicability where clinicians can use these methods on a regular basis, easily interpret the results and be able to explain to their patients and their families what the numbers/images mean. The 3DSS based graphical algorithm presented in this paper provides clinicians with a simple way to quickly and reliably quantify, for the first time, key cosmesis parameters that are already part of routine clinical assessment.

The current study shows that measurements made using the semi-automated tool were reliable. The ICC results for intra-user reliability indicate that familiarity with torso anatomy and scoliosis was necessary for reliable scapula and hip asymmetry measurements. The novice user reported difficulty in identifying scapula prominences, particularly in Lenke type 1 patients where the scapula was often obscured by the rib prominence or in patients with high BMI. Prior training will help improve scapula prominence identification as demonstrated by the higher intra-user reliability in the expert user measurements. However, ShA, TR and HPS were shown to be measured reliably even by a novice. In particular, these measurements, which are the most clinically relevant metrics, showed very high reliability in both intra-user and inter-user assessments.

The workflow presented in this paper has several advantages over current methods. First, the Artec Eva scanner not only has a high geometrical accuracy, but it is also a handheld device and does not require complicated or expensive installation, nor a specific room setup. Second, while some experience in scoliosis is required to correctly place the ROIs for certain metrics, the algorithm itself can be used effectively with minimal instruction, as demonstrated by the novice user. Third, the results obtained from the algorithm are straightforward angles of distinct anatomical features and can be easily interpreted by clinicians and patients alike. Finally, 3DSS combined with this measurement tool provides clinicians a way to store ST information for quick repeat measurements at a later date, similar to how radiographs are handled in clinic when analysing medical history and the trajectory of scoliosis progression across multiple appointments. This convenience is not easily afforded by other optical projection methods and the ability to re-measure old scans can additionally aid in tracking progression and significantly reduce common clerical errors.

The base concept of the semi-automated measurement tool has the potential to be used in several other applications where cosmesis assessments are made. For example, we envision its use in measuring the degree of skeletal/growth deformities other than AIS, in the analysis of symmetry and anatomical alignment in reconstructive surgery (breast, face, etc.) and in tracking the superficial appearance of a healing wound or growing lesion.

The main limitation of this workflow is the scanning procedure itself. Patients were scanned standing with fingertips placed on their shoulders. In addition, to keep the children comfortable during the scanning process, they have, to date, only been required to remove their tops and roll down their pants/skirts below the PSIS. While clothing and undergarments are easily subtracted from the surface mesh during 3D model processing, excessive mesh repair is often required to obtain a clean shoulder and neck region, free from artefacts due to fingertips. The scanning protocol is now optimised to have patients place their index finger in their ears instead of placing their fingertips on their shoulders. Any future scans will thereafter only require minimal processing to obtain a clean mesh, and therefore result in a faster workflow and more reliable shoulder measurements.

## Conclusion

In conclusion, this work has established a streamlined cosmesis assessment workflow that uses a semi-automated algorithm to measure five key cosmesis metrics from patient-specific 3DSS data. The measurement tool was shown to be reliable and readily implemented with minimal instruction required. The quantification of these key AIS cosmetic metrics will enable clinicians to track external torso deformity in a categorical manner alongside clinical metrics obtained from radiography across multiple review appointments during their scoliosis surveillance period.

Future work includes further optimisation of the standard scanning procedure in clinic and workflow streamlining. Correlation with clinical metrics both pre- and post-op are important to quantify external deformity progression and correction parameters. In addition, the TR module of the algorithm will be further developed by being converted to a standalone virtual digital scoliometer that can be used to measure rib prominence and angle of trunk rotation on 3DSS performed in the forward bending position.

## Data Availability

The raw measurement data and statistical analysis made on the algorithm will be made fully available to the public via QUT’s Institutional Research Data Storage Service (RDSS) after the paper has been published. Through the RDSS, noninstitutional researchers may be granted access to this data after making a request to myself as first author (Dr Sinduja Suresh, s.suresh@qut.edu.au) and owner of the RDSS entry. The Grasshopper workflow developed in this project is not yet available for public dissemination because we are currently exploring opportunities with local hospitals to include it in a smart device application. However, the workflow is built on commercially available software, and we have described the functionality in detail. We believe that someone with sufficient experience in Grasshopper could replicate this functionality described in the Methods and Results section. The original 3DSS patient dataset cannot be shared publicly as it may compromise the privacy and confidentiality of our participants.
